# Determination of Monosodium Glutamate in Noodles Using a Simple Spectrofluorometric Method based on an Emission Turn-on Approach

**DOI:** 10.1007/s10895-023-03143-0

**Published:** 2023-01-17

**Authors:** Amira H. Kamal, Samah F. El-Malla, Rehab H. Elattar, Fotouh R. Mansour

**Affiliations:** Department of Pharmaceutical Analytical Chemistry, Faculty of Pharmacy, Tanta University, Elgeish Street, Tanta, 31111 Egypt

**Keywords:** Mono sodium glutamate, Iron (III) salicylate, Spectrofluorometry, Food additives, Ligand exchange, Metal complexes

## Abstract

A simple, fast, and ecofriendly spectrofluorometric method was developed and validated for determination of mono sodium glutamate (MSG). The method depended on the reaction between MSG and iron (III) salicylate based on ligand exchange mechanism. Addition of MSG turned-on the fluorescent response of iron (III) salicylate at λ_em_ 411 nm. Reaction conditions including reagent concentration, pH, and time were optimized. The method was validated regarding the ICH guidelines. The method determined MSG within the linearity range of 25—250 µM with a coefficient of determination of 0.9967 and a calculated limit of detection of 1.73 µM. Furthermore, the developed method was successfully applied for the determination of MSG in food preparation (instant noodles). The results were compared to those obtained by a published HPLC method using t-test and F-test at 95% confidence interval; no statistically significant difference was found. Based on the analytical Eco-scale and the green analytical procedure index (GAPI), the developed method was assessed to be greener than the published HPLC method. The developed method offered advantages over other spectrophotometric reported methods and was convenient for routine determination of MSG in foodstuffs.

**Supplementary Information:**

The online version contains supplementary material available at 10.1007/s10895-023-03143-0.

## Introduction

Monosodium glutamate (MSG) is the sodium salt of glutamic acid; an amino acid that is naturally found in the plants and animals [1]. MSG is one of the commonly used food additives, with a code number of E621, according to European regulations [2, 3]. MSG is also a flavor enhancer to give the unique Umami taste [4] in fast food products, such as instant noodles, soups, sauces, pizza, crisps and potato snacks [5, 6]. MSG is synthesized by different methods including hydrolysis of some vegetable proteins by hydrochloric acid as well as bacterial fermentation or direct chemical synthesis [3, 7, 8]. Unlike natural glutamic acid found in food, synthetic glutamic acid is a harmful substance, especially when it is taken above the permissible concentrations in foodstuffs (0.2 to 0.8%) [9, 10]. The daily dose of MSG should not exceed 0.3–1.0 g [10–12]. MSG has a delicious taste, which urges eating more amount of food and helps with anorexia [13]. Excess MSG in food may lead to Chinese restaurant syndrome with symptoms of headache, weakness, chest pain, flushing, nausea as well as heart palpitations [14]. MSG was associated with diabetes mellitus (Type II) in experimental models [15]. High doses of MSG in small rodents induce obesity, increase fat mass and damage body organs such as the kidney liver, thymus and brain [16]. These detrimental effects could be due to the glutamate-induced oxidative damage. Studies revealed that excessive MSG can lead to neurotoxic effects, acute degenerative changes and sudden death of neurons [17]. In addition, it has been reported that high doses of MSG in the neonatal period can induce learning and memory impairment, affecting the nervous system, kidney functions, and vision [16, 18]. These reports explain why the determination of MSG is very important.

Several methods were reported for determination of MSG, including UV/visible spectrophotometry [10, 19–23]**,** spectroflourometry [24–26]**,** and electrochemistry [27–30]. Other methods were reported for separation of MSG including capillary electrophoresis [31], paper chromatography [32], thin layer chromatography [33], LC–MS/MS [34] and HPLC with UV detection [35, 36], fluorescence detection [37] and evaporative light scattering detection [38]. Because MSG lacks a fluorophore, derivatization reaction is required for its determination, but these reactions need expensive reagents and specified conditions such as prolonged reaction time, heat, and catalysis.

The purpose of this work is to develop a rapid and simple spectrofluorometric method for detection of MSG in noodles. The method relied on turning-on salicylate fluorescence after ligand exchange reaction of iron (III) salicylate and MSG. The response of salicylate emission was directly proportional to the concentration of MSG. To the best of our knowledge, MSG could be used for determination of some metals such as iron and cupper but this is the first spectrofluorometric reaction that is developed for analysis of MSG in food samples using metal complexes [39, 40]. Advantages of fluorescence spectrophotometry over other analytical techniques are that it is more sensitive and specific than UV/Vis spectrophotometry and does not require the sophisticated instrumentation of chromatographic techniques [41]. Fluorescence spectrophotometry is more selective than UV/Vis spectrophotometry because not all molecules that absorb UV/Vis radiation will fluoresce. Furthermore, fluorescence measurements rely on two wavelengths (excitation and emission), whereas UV/Vis spectrophotometry relies on only one wavelength. It is certainly less likely that another compound will have similar excitation and emission wavelengths. This makes spectrofluorometry more selective than UV/Vis spectrophotometry [42]. The developed method is green, simple, fast, and does not require any expensive reagents or complicated processes and is convenient for routine detection of MSG. The principles of green analytical chemistry (GAC) emphasize the use of environmentally friendly and sustainable techniques in the development and implementation of analytical methods. The method greenness was assessed by the Green Analytical Procedure Index (GAPI) and analytical Eco-scale, and the scores were compared with those of a reference method.

## Materials and Methods

### Apparatus and Software

A JASCO spectrofluorometer (model FP‐6300, Tokyo, Japan) was used. It equipped with holographic grating (1500 grooves/mm) and modified Rowland mount excitation and emission monochromators in addition to 150 W xenon lamp. Spectra Manager Software V1.53.01 was used. The slit widths were 10 nm and 5 nm for excitation and emission monochromators, respectively. The scanning speed was 1000 nm/ min. The sensitivity was low and the response was medium. The wavelength of maximum excitation (λ_ex_) was 290 nm and that of maximum emission (λ_em_) was 411 nm. A quartz cuvette was used (path length 1 cm). Automatic Water Still (Sci Finetech, Seoul, South Korea) and Sartorius AG digital analytical balance (Germany) were used.

### Chemicals

MSG (99%, purity) was kindly supplied by Sigma Pharmaceutical Industries (Quesna, El-Menoufia, Egypt). Sodium salicylate (99.8%, purity) (Dop Organic Kimyasan, Istanbul, Turkey), nitric acid (Merck, Darmstadt, Germany), ammonium iron (III) sulphate (98.5%, purity) (Alpha Chemia Lab reagent), HCl (37%w/w) purchased from Fisher scientific (Geel, Belgium) and NaOH (99%, purity) purchased from ADWIC Co (Al-Qalyubia, Egypt) were used.

### Reagents and MSG Standard Solutions

A diluent solution containing 0.25 M nitric acid was prepared by transferring 1.593 mL of nitric acid (Concentration) into 100-mL volumetric flask, and then diluting to volume with distilled water. 10 mM iron (III) salicylate standard solution was prepared by mixing 482 mg of ammonium iron (III) sulfate (482.19 g mol^−1^) and 160 mg of sodium salicylate (160.1 g mol^−1^) then diluting to 100 mL with the diluent to enhance the stability of the iron (III) salicylate complex [43, 44]. A working reagent solution of 1 mM iron (III) salicylate was prepared by diluting 10 mL of the previously prepared standard solution to 100 mL with distilled water.

MSG standard solution (50 mM) was prepared by weighing 0.423 g of MSG (169.111 g mol^−1^) and dissolving in 50 mL distilled water. A working solution of 5 mM MSG was prepared by diluting 10 mL of the standard solution to 100 mL with distilled water.

### Reaction Procedures and Calibration Curve

Different aliquots of MSG working standard solution (50 to 500 μL) were transferred into 10-mL volumetric flasks to prepare serial dilutions in the concentration range of 25 to 250 μM. To each flask, 700 µL of 1 mM iron (III) salicylate reagent was added; the volume was completed to the mark by distilled water. Then, fluorescence spectra were recorded immediately. The emission spectrum of reagent blank was also recorded. The relative fluorescence spectra were calculated by subtracting the blank fluorescence spectrum from that of the MSG reaction solutions. The calibration curves (n = 3) were constructed by plotting the relative increase in fluorescence intensity at 411 nm versus the final concentrations of MSG in µM and regression equation was calculated.

### Determination of MSG in Instant Noodles and Chinese Salt

One packet of instant noodles contains a sachet of a seasoning powder weighing 4.7099 g. Noodles stock solution was prepared by weighing 0.5 g of the seasoning powder, to be transferred to 25-mL volumetric flask, dissolved in 20 mL distilled water, sonicated for 10 min, completed to the mark with distilled water, filtered and then 1 mL of the filtrate was diluted to 25 mL with water to prepare a working sample solution. Various volumes (50, 100, 150, 200, 250, 300, 350, and 400) µL of MSG working standard solution (5 mM) were added to 1 mL of the instant noodles working solution into 10-mL volumetric flasks. Then the reaction was performed, as described in the previous section. Standard addition curves (n = 3) were generated, and regression equation was calculated. The amount of MSG in the final sample solution (μM) was calculated at the X-intercept of the standard addition line and then multiplied by the factor of 9.956 to calculate the final concentration of MSG in mg/sachet.

One packet of Chinese salt contains 99% MSG. The salt stock solution was prepared by weighing 0.42 g of the salt, completed to 50-mL distilled water in volumetric flask. The working standard solution was prepared by taking 10 mL from the stock solution completed to 100 mL distilled water in volumetric flask. Then the reaction was applied with 0.3 mL from the working standard solution and the concentration of MSG was determined.

### Reaction Stoichiometry

The mole ratio method was employed to determine the stoichiometric ratio of the reaction between MSG and iron (III) salicylate [45]. Different standard mixture solutions containing iron (III) salicylate reagent and MSG were prepared by adding different concentrations of MSG (50–800 µM) into a series of 10-mL volumetric flasks containing 70 µM iron (III) salicylate. The mole ratio graph was obtained by plotting the relative fluorescence intensity (ΔF_411nm_) against the mole ratio ([MSG] / [iron (III) salicylate]). As shown in Fig. S1, the reaction ratio was found to be 4:1 to (MSG: iron (III)-salicylate).

## Results and Discussion

### Reaction Mechanism

**Fig. 1:**
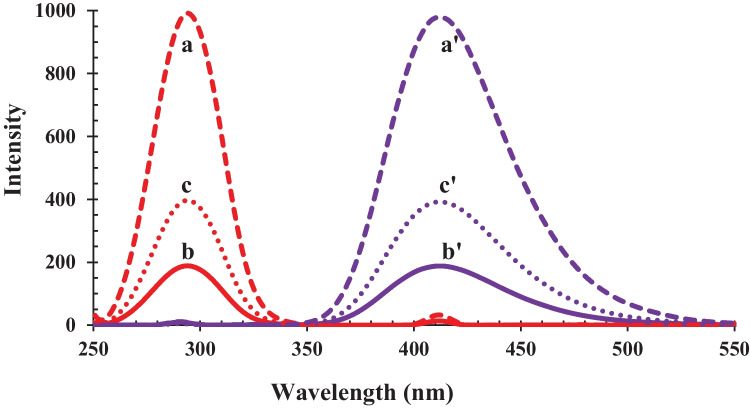
Excitation (Red Lines) and emission (Blue Lines) spectra of salicylate (**a**, **a'**), iron (III) salicylate before (**b**, **b'**) and after (**c**, **c'**) addition of MSG

MSG contains a weak chromophore (-C = O), which makes its determination by direct spectrofluorometry not possible as it does not have the essential conjugated rigid structure (fluorophore). In this work, a new spectrofluorometric method for determination of MSG, using iron (III) salicylate was developed. Salicylate is a fluorescent substance but when it forms a complex with iron (III), the fluorescence of salicylate is turned-off (Fig. 1a, b). The quenching effect produced is due to the paramagnetic properties of iron (III) that induces non-radiative intersystem crossing and triplet states formation [41, 44]. In this reaction, MSG can complex with iron (III) leading to liberation of free salicylate due to ligand exchange reaction. Fig. S2 illustrates the proposed mechanism of the reaction between MSG and iron (III) salicylate**.** The emission of salicylate was turned-on when MSG was added, as shown in Fig. 1c, with a proportional increase in fluorescence with the increase in MSG concentration.

### Method Optimization

The influence of factors that could affect the reaction was studied including reagent concentration, pH, and reaction time. Fluorescence intensity was determined in the presence and in the absence of MSG and *ΔF*_*411 nm*_ was calculated. The optimum values of these parameters were those achieving the highest *ΔF*_*411nm*_ for attaining the highest possible sensitivity.

**Fig. 2:**
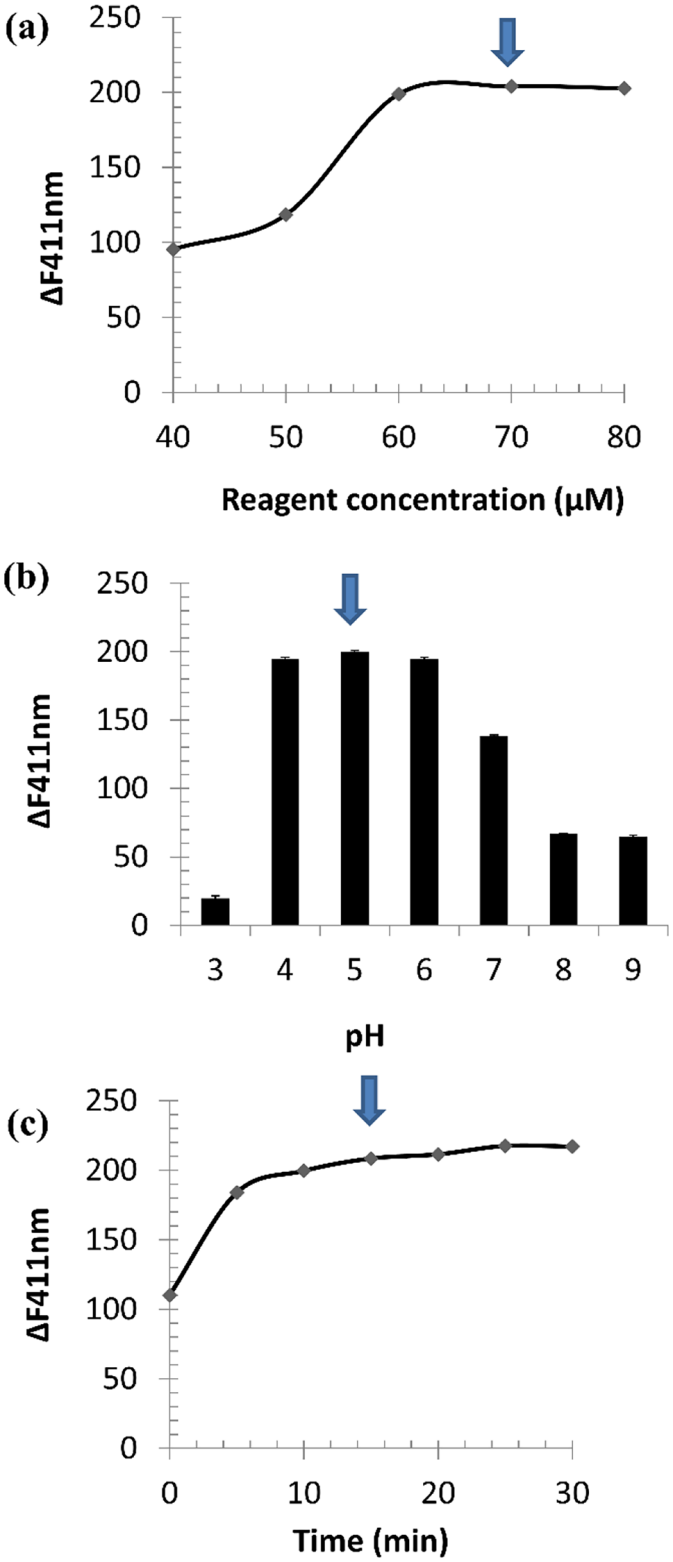
Effect of iron (III) salicylate concentration **a**, pH **b** and time **c** on *ΔF*_*411nm*_

The influence of iron (III) salicylate concentration was investigated by performing the reaction using different concentrations of the reagent (40 to 80 µM), as shown in Fig. 2a. It was found that ΔF_411nm_ increased by increasing the reagent concentration up to 60 µM, and then a plateau was observed. The concentration of 70 µM iron (III) salicylate was selected to be the optimum reagent concentration.

Evaluation of the optimum pH for the reaction was performed by diluting the reaction flasks with water adjusted to suitable pH with 0.05 N HCl and /or 0.05 N NaOH to prepare diluents in the pH range of 3 to 9. MSG has three pKa values: 4.07 (ω-COOH), 2.10 (α-COOH), and 9.47 (α-NH_2_). It was found that MSG-complex stability was very poor at pH 3. This could be attributed to the incomplete ionization of α-COOH and ω-COOH, which were needed for the chelation process. Maximum *ΔF*_*411nm*_ was obtained in the pH range of 4 to 6 which explains the high stability of the formed MSG-complex when both -COOH groups in MSG are ionized. This ensures that both carboxylate groups are involved in chelation with Fe (III) as shown in Fig. S2. At pH values higher than 6, *ΔF*_*411nm*_ was decreased, which could be attributed to precipitation of Fe(OH)_3_. The optimum pH was selected to be at pH 5, as shown in Fig. 2b. The reaction was also performed by diluting with water, and no difference in *ΔF*_*411nm*_ value was observed when compared with the reaction performed at pH 5, so water was selected as the best solvent with no need for pH adjustment.

Reaction time was studied from 0 to 30 min. Ligand exchange occurred instantaneously with a gradual increase in *ΔF*_*411nm*_ with time, upto 70 min, after which the response was relatively stable (Fig. 2c). The optimum reaction time was selected to be 15 min, to guarantee reaction completion before measurements.

### Method Validation

The proposed method was validated as per the guidelines of the International Conference on Harmonisation (ICH) in terms of linearity, range, limit of detection (LOD), accuracy, precision and robustness (ICH Q2A, 1995).

**Fig. 3:**
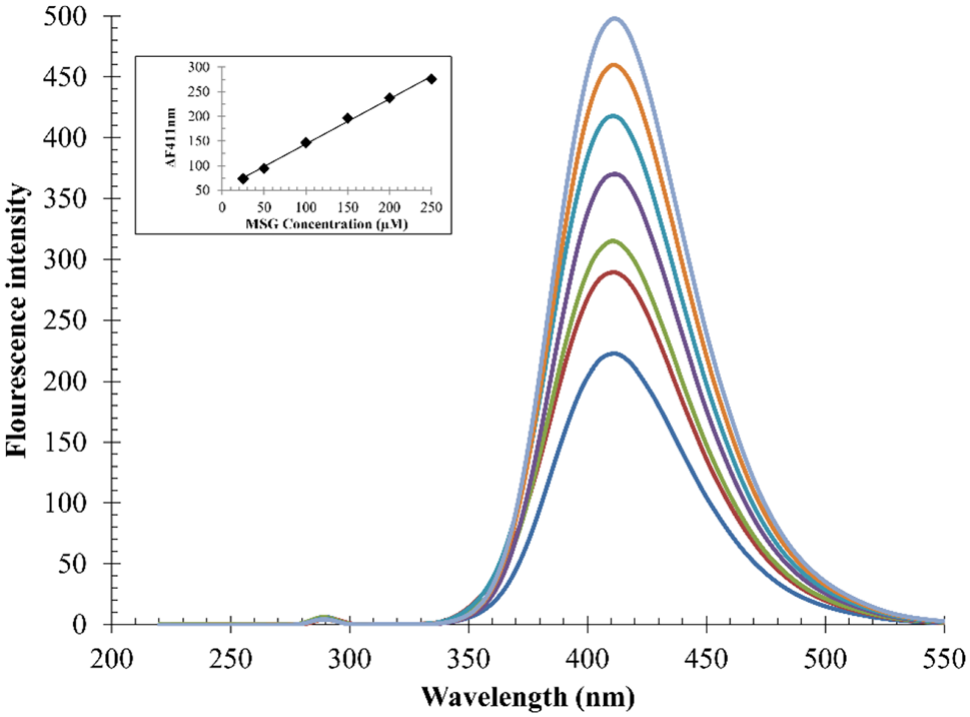
Fluorescence spectra obtained in presence of different concentrations of MSG (0, 25, 50, 100, 150, 200 and 250 μM from down top) to 70 μM iron (III) salicylate. Figure inset: calibration curve of MSG

Linearity of the developed method was estimated by plotting *ΔF*_*411nm*_ versus the concentration of MSG (µM), as shown in Fig. 3. Regression analysis was performed using SPSS (SPSS Chicago, IL, USA, Version 26), and the correlation coefficient was found to be 0.998. Table 1 shows the statistical data of the regression equation. The small values of the standard deviations about intercept (S_a_), slope (S_b_), and residuals (S_y/x_) indicate the acceptable linearity over the concentration range 25–250 μM. Limit of detection (LOD) was determined according to ICH guideline Q2 (R1), using the standard deviation of the blank signal (SD _blank_) and the slope of the calibration curve (b) as follows:

**Table 1:** Regression parameters for analysis of MSG

**Range (µM)**	**r**	**a**	**b**	**S**_a_	**S**_b_	**S**_y/x_	**LOD (µM)**
25–250	0.998	52.57	0.915	4.01	0.026	5.16	1.73

$$\mathrm{LOD}=3.3\times {\mathrm{SD}}_{\mathrm{blank}}/\mathrm{ b}$$

The calculated LOD was found to be 1.73 μM (Table 1) which indicated that the method had sufficient sensitivity for determination of MSG in bulk, diluted solutions, and food samples.

a, intercept; r, correlation coefficient; LOD, limit of detection (calculated).b, slope; S_a_, standard deviation of intercept; S_y/x_, standard deviation of residuals; S_b_, standard deviation of slope;

The accuracy of the developed method was assessed by triplicate determination of three different concentrations of standard MSG within the linearity range and the % recovery was calculated (Table S1). The reasonable value of % recovery (100 ± 2%) with small value of standard deviation (SD < 2) demonstrated the method accuracy. The precision of the method was evaluated by testing the intra-day and the inter-day precision. The intra-day precision was evaluated by determination of three replicates of three different concentrations of MSG covering the linearity range in the same day. Similar procedures were repeated for assessing inter-day precision in three consecutive days. The standard deviation (SD) and the percent relative standard deviation (% RSD) was calculated (Table 2). Small values of % RSD (less than 2%) confirmed the precision of the developed method.

**Table 2:** Evaluation of the precision of the developed method

**Concentration taken (μM)**	**Intra-day precision**			**Inter day precision**		
	Concentration found^*^ **(μM)**	**SD**	**%RSD**	Concentration found^*^ **(μM)**	**SD**	**%RSD**
50.00	49.84	0.33	0.663	49.75	0.242	0.487
125.00	125.02	0.718	0.574	125.01	0.277	0.221
200.00	199.77	0.429	0.215	199.69	0.105	0.0526

*Average for three determinations, RSD: relative standard deviation, SD: standard deviation

To verify specificity of the developed method for spectrofluorometric determination of MSG in the food samples. The effect of some substances including glucose, lactose, starch, NaCl, KCl and glycine were studied. It was observed that there is no interferences upon high concentrations of these substances (Table S2). So that this developed method has a good specificity for quantitation of MSG

The robustness of the developed method was evaluated by studying the effect of small deliberate changes in the experimental parameters on the fluorescence response and % recovery. The studied variables included the reagent concentration (70 ± 5 µM), emission wavelength (411 ± 2 nm) and time (15 ± 3 min). The small values of % RSD as shown in (Table 3) indicated that the robustness of the method.

**Table 3:** Assessment of the robustness of the developed method

**Variable**	**value**	**% Recovery**	**Mean % Recovery**	**SD**	**% RSD**
Reagent concentration (µM)	65 70* 75	100.33 100.26 100.24	100.27	0.046	0.047
Wavelength (nm)	409 411* 413	100.07 100.00 100.31	100.13	0.162	0.162
Reaction time (min)	12 15* 18	100.98 100.29 100.04	100.44	0.483	0.481

*Optimum condition

### Application to Real Food Samples

The developed method was applied for the detection of MSG in instant noodles and Chinese salt. Three replicates were measured, and the mean concentration found in noodles and in Chinese salt were calculated. The results obtained were statistically compared with those acquired by a reported HPLC method [46]. The comparison method was based on gradient HPLC analysis after pre-column derivatization with 2,4-dinitrofluorobenzene (DNFB) for simultaneous determination of amino acids in tea infusion. Student *t*-test and *F*-test at (p-value 0.05) were applied. The difference in the calculated mean concentration or in the SD was not statistically significant and good agreement was achieved (Table 4).

**Table 4:** Assay of MSG in instant noodles and Chinese salt by the developed and reported methods

**Food sample**		**Developed method**	**Reported HPLC method **[46]
	**Mean**^a^ **±** **SD**	681.86 ± 1.32	682.54 ± 2.05
**Noodles**	**t-test**	0.34	(2.776)^b^
	**F-test**	2.42	(19)^b^
	**Mean**^a^ **±** **SD**	100.05 ± 0.40	99.66 ± 1.23
**Chinese salt**	**t-test**	0.39	(2.78)^b^
	**F-test**	4.73	(19)b

^a^Mean concentration was calculated for three determinations(mg/packet) for noodles and (% w/w) for Chinese salt

^b^Tabulated value for t-test (0.05) and F-test (0.05)

### Greenness Assessment

**Fig. 4:**
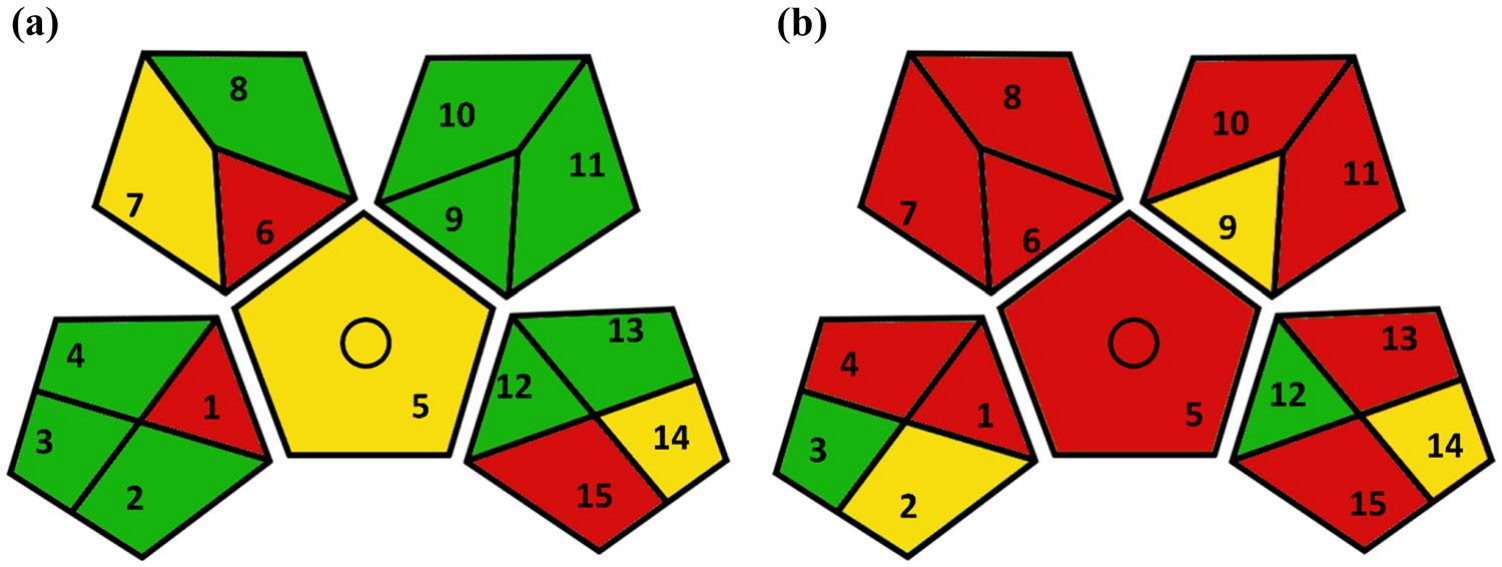
GAPI of the developed spectrofluorometric (**a**), and the reported HPLC (**b**) methods using GAPI

Green analytical chemistry (GAC) principles and the development of ecofriendly analytical method have gained interest in the past few years. Different tools were developed for evaluating the greenness of analytical methods, e.g., Green Analytical Procedure Index (GAPI) and analytical Eco-scale. The concept of analytical Eco-scale depends on assigning penalty points (PPs) for each parameter such as amount/type of reagents, instruments, waste and hazards. The sum of these PPs are subtracted from the total value of 100 which represents the ideal green analysis to calculate the method’s score [47]. On the other hands, GAPI depends on assigning colors (green, yellow, and red) to five pentagrams representing the following parameters: sample collection/preservation/transport/storage, sample preparation, reagent/solvents, instrumentation, and type of analytical method (qualitative and/or quantitative). Color codes represent the level of greenness of the analytical method [48]. Both tools were applied for assessment of greenness of the developed spectrofluorometric method and compared to that of the reported HPLC method. Analytical Eco-scale score of 88 (> 75%) (Table 5) and high density of green color in GAPI pictogram proved the excellent greenness of the developed method, as illustrated in Fig. 4.

**Table 5:** Analytical Eco Scale assessment of the greenness of the presented and the reported methods using

**The developed Method**		**The reported HPLC method** **[**46**]**	
Reagents	PPs*	Reagents	PPs*
NH_4_Fe(SO_4_)_2_ (< 10 g)	1	DNFB (< 10 mL)	8
		NaHCO_3_ (< 10 g)	1
Sodium salicylate (< 10 g)	1	KH_2_PO_4_, CH_3_COONa (< 10 g)	0
		HCl (< 10 mL)	4
HNO_3_ (< 10 mL)	4	Methanol (10–100 mL)	12
		THF (< 10 mL)	6
**Ʃ**	6	**Ʃ**	31
**Instruments**	**PPs**	**Instruments**	**PPs**
Spectrofluorometer	0	HPLC	0
Occupational hazards	0	Occupational hazards	3
Waste (˂10 mL, No treatment)	6	Waste (˂10 mL, No treatment)	6
**Ʃ**	6	**Ʃ**	9
**Total**	12	**Total**	40
**SCORE: 88**		**SCORE: 60**	

*Calculations of penalty points (PPs) were performed per sample, DNFB: dinitrofluorobenzene, THF: tetrahydrofuran

Total PPs = Ʃ_PPs, reagents_ + Ʃ _PPs, instruments_. SCORE = 100 – total PPs

### Comparison with Other Reported Methods

Direct spectrofluorometric detection of MSG is not possible due to lack of fluorophore. It is a challenge to develop a simple spectrofluorometric method for MSG analysis. Most of the reported spectrofluorometric methods used enzymes for the determination of MSG in multi-step reactions [24]. Despite being highly sensitive, these enzymatic methods require expensive kits and need special precautions to maintain the stability of enzymes. Other reported methods employed derivatizing agents, such as fluorescamine as a fluorogenic label. In this method, MSG was determined at the zero-crossing point of aspartame using the iso-differential synchronous derivative spectrofluorometry. However, this method showed poor precision (high % RSD) which may be attributed to analysis at sloppy positions in the derivative spectra [25]. Dansyl hydrazine was also used for simultaneous determination of MSG and aspartic acid in presence of dextran. MSG binds to dextran, quenching the fluorescence response of dextran due to the inhibition of the charge transfer. However, other amino acids that could interact with dextran could interfere with MSG measurement [26]. Compared with other published spectrofluorometric methods (Table 6), our developed method is easier, faster, more ecofriendly, and does not require any difficult processes or special apparatus. The ligand exchange reaction involved in this method is a single step process performed at ambient temperature. The achieved sensitivity of the developed method was sufficiently suitable for detection of MSG in foodstuffs. The simplicity of procedures and the high precision made this method preferable over other methods, and more convenient for routine work.

**Table 6:** Comparison between the presented method and the reported spectrofluorometric methods for MSG analysis

**Derivatizing reagent**	**Solvent**	**Time**	**Wavelength** **λ**_ex_**/** **λ**_em_ **(nm)**	**Linearity range (µg/mL)**	**Sample**	**Comments**	**Ref**
NAD^+^, GDH	Glycine hydrazine buffer	30 min	340/450	1.69 × 10^–5^ ‒50.7 × 10^–5^	Biological sample	• Reagents must be freshly prepared and stored properly to ensure short term stability • Reactions involve more than one step • Expensive enzymes and substrates are needed	[49]
Thionine, NAD^+^, GDH	Phosphate buffer pH 7 ± 0.1	20 min	590/620	0‒135.28	Food & blood samples		[24]
Fluorescamine	Phosphate buffer pH 9, acetone	–	393*	0.1‒1	Commercial dried soap	• Acetone is used as solvent for reagent	[25]
Dansyl hydrazine attached to dextran	1% Phosphate buffer	….	275/475	16.9‒4227	Biological sample	• Several reagents are used for preparation of DD so time is consuming • Decomposition of DD may be occurred	[26]
Iron (III) salicylate	Water	–	290/411	4.22‒42.27	Instant noodles	• Simple, rapid, and instantaneous reaction • Water is used as a solvent	This method

GDH l-Glutamate dehydrogenase, NAD^+^ Nicotinamide adenine dinucleotide

*Synchronous derivative spectrofluorometry

## Conclusion

A new switch-on spectrofluorometric method has been developed for determination of MSG in foodstuffs with acceptable accuracy and precision. Addition of MSG to iron (III) salicylate reagent liberates the fluorescent free salicylate after inducing ligand exchange reaction. Compared with the other spectrophotometric methods used for the determination of MSG, this reaction is instantaneous and does not require heating or catalysis. The method was found greener, simpler and faster than the other reported HPLC and spectrofluorometric methods. This principle can be applied for other weak chromophoric molecules that are able to form stable complexes with Fe^3+^. The developed method can be easily applied in food testing laboratories in food industry for the routine analysis of MSG. Additional sample treatment using the recent preparation techniques such as solid and liquid microextraction are expected to improve the sensitivity, and to make the method applicable for determination of very low concentrations of MSG in biological fluids. Extending this work to include other metal-complexes for indirect determination of pharmaceutically active compounds, supplements or food ingredients is the focus of our future research.

## Supplementary Information

Below is the link to the electronic supplementary material.

Supplementary file1 (DOCX 176 KB)

## Data Availability

The datasets generated and analyzed during the current study are available in the supplementary material.
