# Supplementary material for: Determination of Monosodium Glutamate in Noodles Using a Simple Spectrofluorometric Method based on an Emission Turn-on Approach
Source: J Fluoresc. 2023 Jan 17;33(4):1337–46. doi: 10.1007/s10895-023-03143-0 (PMC10361856; doi:10.1007/s10895-023-03143-0)
Supplement: Supplementary file 1 — Supplementary file1 (DOCX 176 KB) [file 10895_2023_3143_MOESM1_ESM.docx]

Determination of monosodium glutamate in noodles using a simple spectrofluorometric method based on an emission turn-on approach

Amira H. Kamal, Samah F. El-Malla, Rehab H. Elattar, Fotouh R. Mansour^*^

*Department of Pharmaceutical Analytical Chemistry, Faculty of Pharmacy, Tanta University, Tanta, Egypt, 31111*

**Fig. S1:** Mole ratio method for determination of the stoichiometry of the reaction between MSG and iron (III) salicylate.





**Fig. S2:** The proposed mechanism of the reaction between MSG and iron (III) salicylate**.**


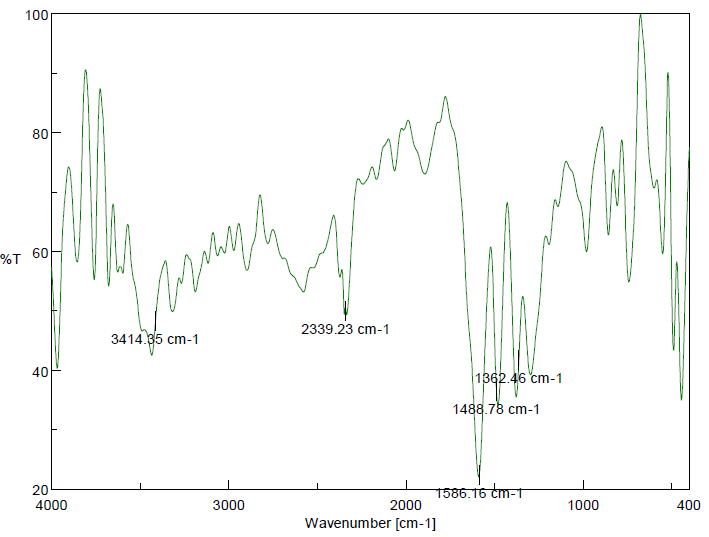


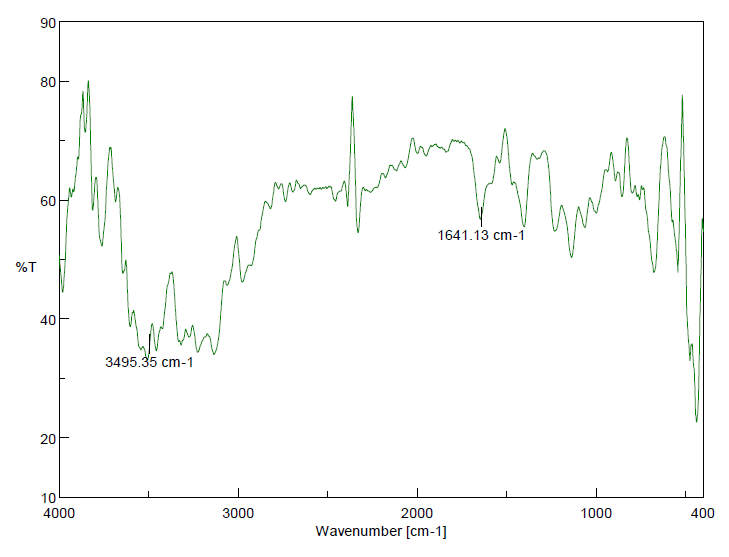


**Figure S3:** IR spectrum of a- sodium salicylate, b- iron (III) salicylate

**Table S1:** Accuracy evaluation for the developed method.

| **Concentration taken (μM)** | **Concentration found**  **(µM)** | | | **Mean concentration found (μM)** | **% Recovery** | **Average % Recovery ± SD** |
| --- | --- | --- | --- | --- | --- | --- |
| **50** | 49.62 | 49.27 | 49.54 | 49.48 | 98.96 | 99.65± 0.28 |
| **125** | 125.54 | 125..0 | 124.99 | 125.28 | 100.22 |  |
| **200** | 199.42 | 199.29 | 199.99 | 199.57 | 99.78 |  |

SD: standard deviation**.**

**Table S2:** Specificity evaluation for the developed method.

| Interfering Substances | Tolerated concentration (mg/mL) |
| --- | --- |
| Glucose | 4.00 |
| Lactose | 1.50 |
| Starch | 1.00 |
| NaCl | 3.20 |
| KCl | 3.20 |
| Glycine | 0.25 |
